# A Technology-Aided Program to Help People With Intellectual and Multiple Disabilities Access Leisure Stimuli and Engage in Cognitive and Physical Activity: Development and Usability Study

**DOI:** 10.2196/82596

**Published:** 2025-10-15

**Authors:** Giulio E Lancioni, Gloria Alberti, Chiara Filippini, Nirbhay N Singh, Mark F O’Reilly, Jeff Sigafoos

**Affiliations:** 1 Lega F. D'Oro Research Center Osimo Italy; 2 College of Medicine Augusta University Augusta, GA United States; 3 Department of Special Education University of Texas at Austin Austin, TX United States; 4 Faculty of Education, Health, and Psychological Sciences Victoria University of Wellington Wellington New Zealand

**Keywords:** intellectual disability, deafness, touch screen technology, leisure, cognitive activity, physical exercise

## Abstract

**Background:**

People with moderate to severe intellectual disability can have difficulties accessing leisure stimuli and engaging in basic cognitive and physical activity independently. These difficulties may be even more marked in individuals with a combination of intellectual disability and sensory or sensory-motor impairments.

**Objective:**

This study assessed a new program relying on touch screen technology, which was set up to support access to leisure stimuli and the performance of a simple form of cognitive activity and basic physical exercise for adults with intellectual or intellectual and hearing disabilities, lack of functional speech, and poor motor dexterity.

**Methods:**

The program alternated access to preferred stimuli (ie, songs, comic sketches, or cartoons) with cognitive activity (ie, matching-to-sample tasks) and physical exercise (ie, body movements). The touch screen technology was modified to ensure that people with poor motor dexterity would be effective in their responding regardless of the accuracy of their responses. The program was implemented with 7 participants. Its impact was assessed through the use of single-case research methodology.

**Results:**

During the baseline (when standard technology was used), the mean percentage of songs, comic sketches, or cartoons accessed; match-to-sample responses provided; and body movements performed correctly and independent of research assistants’ help was 0% for all participants with a single exception. During the intervention (when the new program with modified touch screen technology was used), the participants’ mean percentage of songs, comic sketches, or cartoons accessed correctly and independent of research assistants’ help per session was virtually 100%. Their mean percentage for correct match-to-sample responses provided and correct body movements performed independent of research assistants’ help was within the 90% to 100% range.

**Conclusions:**

The findings suggest that the program may constitute a useful tool for helping people with intellectual and multiple disabilities access leisure stimuli and engage in cognitive and physical activity.

## Introduction

### Background

People with moderate to severe intellectual disability can have difficulties accessing leisure stimuli and engaging in basic cognitive and physical activity independently [1-7]. For example, their reduced manual dexterity and unfamiliarity with technological gadgets may limit their opportunities to operate standard devices available to select and activate preferred leisure stimuli, such as songs, comic sketches, and cartoons [8-12]. Their tendency to remain fairly isolated and their low motivation and self-determination may interfere with their possibility to (1) engage in cognitive activities such as listening to and answering questions related to their interest areas or performing stimulus discrimination and stimulus matching tasks [11-16] and (2) perform simple, beneficial body movements involving the arms, legs, or head and neck [17-20]. These difficulties may be even more marked in individuals with a combination of intellectual disability and sensory or sensory-motor impairments (eg, hearing loss and reduced ambulatory skills) [11,21-25].

The use of staff supervision to help people with intellectual and multiple disabilities access leisure options and practice cognitive activities and physical exercise may present 2 drawbacks. First, such supervision would be rather costly in terms of staff time and not always feasible in contexts in which staff availability is limited [3,7]. Second, the use of staff supervision would be unlikely to promote participants’ independence [3,7,10,11]. A possible alternative to staff supervision may be the use of technology-aided programs [26-30]. A variety of technology-aided programs have been developed over the years to enable people to independently perform multistep tasks [3,6,7,31-33], engage in physical exercise [34-38], and produce verbal (easy-to-understand) requests for preferred events [39-45].

Programs have also been developed that have the objective of helping people engage in combinations of activities. The activities combined could include leisure and communication via telephone calls or messages [46]; leisure, communication, and occupational (functional) tasks [47]; or leisure, communication, and cognitive exercises via stories and related questions or series of questions [11,48]. In the last one of those studies [48], a program was assessed with 6 adults who were diagnosed with moderate intellectual disability, motor impairments, and lack of (functional) speech. The program relied on a tablet fitted with internet connection, a SIM card, WhatsApp Messenger, and the MacroDroid app and interfaced with pairs of response sensors. Every session started with the tablet presenting music and a telephone image and asking the participants whether they wanted to listen to music or call somebody. If the participants activated the sensor in front of the music image, the tablet presented the photos of 2 singers. Activating the sensor in front of one of the singers led the tablet to play a song by that singer for 1.5 minutes. The end of the song led the process to be repeated once or twice more. If the participants chose the telephone image, the tablet presented the photos of 2 preferred persons. Choosing one of them led to the start of a video call with that person.

Following the choice period, the tablet presented a series of 5 questions suitable to the participants’ skills. For example, the tablet could present the image of a girl and the image of another person and ask, “Which one is a girl?” If the participants answered correctly by activating the sensor in front of the right image, the tablet provided verbal approval followed by the next question. If participants were incorrect, the tablet remained idle. Once all the questions had been answered, a new choice period started. A session included 4 choice periods and 3 series of questions. The results showed that, during the baseline, the participants were unable to use a standard tablet. During the intervention, they were successful in accessing songs, making video calls, and answering questions.

In light of (1) the encouraging results obtained with the program described previously and other programs targeting combinations of activities and (2) the positive implications of those results for participants and rehabilitation contexts, new research efforts in this area would appear quite relevant. Those efforts should be aimed at designing and evaluating new technology-aided programs for enabling people with intellectual and multiple disabilities to engage in different forms of activities (ie, activities promoting their enjoyment and vitalizing their intellectual and physical functions) independent of staff supervision.

### Objectives

This study assessed a new program relying on touch screen technology that was designed to support access to leisure stimuli and the performance of a simple form of cognitive activity and basic physical exercise for adults with intellectual or intellectual and hearing disabilities, lack of functional speech, and poor motor dexterity. The program alternated access to preferred stimuli (ie, songs, comic sketches, or cartoons) with cognitive activity (ie, matching-to-sample tasks) and physical exercise (ie, series of body movements). The alternation was intended to motivate the participants to successfully manage the latter (presumably less preferred) forms of engagement [49,50]. The touch screen technology was modified to ensure that people with poor motor dexterity would be successful in their responding regardless of the accuracy of their responses. The program was implemented with 7 participants. Its impact was assessed through the use of single-case research methodology.

## Methods

### Participants

Table 1 lists the 7 participants involved in the study using pseudonyms and reports their chronological ages and their Vineland age equivalents obtained using the second edition of the Vineland Adaptive Behavior Scales [51,52]. The participants had a diagnosis of congenital or perinatal encephalopathy with intellectual disability. Three of them (Adriel, Lilah, and Wyatt) also presented with hearing loss, and one of these 3 (Wyatt) was nonambulatory. All participants had functional vision with or without glasses correction but lacked functional speech (ie, could only emit a few sounds or words) and had poor motor dexterity. Their chronological ages ranged from 24 to 64 years. Their Vineland age equivalents varied between 2 years and 3 months and 5 years and 9 months for daily living skills (the *personal* subdomain) and between 3 years and 1 month and 4 years and 8 months for receptive communication. No IQ scores were available for them. Psychological records of the rehabilitation and care centers that they attended reported their level of intellectual disability to be in the moderate or moderate to severe range.

The participants were recruited through direct contact with the centers that they attended. Their selection was based on a number of conditions, which were verified through preliminary observations or staff interviews. First, they did not access preferred stimuli and did not engage in forms of cognitive activity and physical exercise without staff supervision. Second, they were considered unfit to use standard technology because they had difficulties following the multiple operational steps required for that and because they also had difficulties in activating a touch screen monitor given their poor motor dexterity and erratic response patterns (eg, heavy pointing, tapping, and touching with the fingernails). Third, they seemed to enjoy music, comic sketches, or cartoons and, thus, were thought to be strongly motivated to access these stimuli. Fourth, they could engage in basic forms of cognitive activity, such as carrying out simple match-to-sample tasks with various types of stimuli, when staff arranged those tasks for them. Fifth, they were able to imitate body movements considered to be suitable forms of physical exercise (eg, raising their arms and bringing them down to their knees) both when shown by a person in front of them and when presented on a computer screen. Sixth, staff considered the program and the technology used for it (shown to them in advance) functional for the participants and supported the study.

**Table 1:** Participants’ chronological ages and Vineland age equivalents for daily living skills (personal subdomain; DLSP) and receptive communication (RC).

Participant pseudonym	Chronological age (y)	Vineland age equivalent^a^	
		DLSP	RC
Paisley	61	2 years 3 months	4 years 3 months
Adriel	51	4 years 7 months	3 years 1 month
Lilah	63	4 years 11 months	3 years 8 months
Jade	64	5 years 9 months	4 years 3 months
Lainey	48	4 years 4 months	4 years 3 months
Jayden	24	4 years 2 months	3 years 4 months
Wyatt	49	2 years 3 months	4 years 8 months

^a^Age equivalents are based on the Italian standardization of the Vineland Adaptive Behavior Scales [51].

### Ethical Considerations

Regular staff members and research assistants showed the participants the technology (ie, touch screen monitor and the options it contained) and asked them whether they would like to use it. Their positive vocal or behavioral (head nodding) response to the question was taken as consent to the study. However, to make their recruitment highly transparent, their legal representatives were involved in the consent process. Specifically, the legal representatives were asked to read and sign a written consent form on the participants’ behalf authorizing their inclusion in the study. No compensation was available for the participants whose privacy was protected through de-identification. This study was approved by the ethics committee of the Lega del Filo D’Oro, Osimo, Italy (P031020252). All procedures performed were in accordance with the national and international ethical standards and with the 1964 Declaration of Helsinki and its later amendments or comparable ethical standards.

### Setting, Sessions, and Research Assistants

Quiet rooms at the rehabilitation and care centers that the participants attended constituted the setting for this study. This study included baseline and intervention sessions. The baseline sessions were carried out without the use of the technology system developed for this study. The intervention sessions involved the use of the technology system. The sessions were implemented by research assistants, typically once a day, 4 to 6 days a week. Four research assistants were employed to carry out the sessions and collect the data. They held university psychology degrees, had experience applying technology-aided intervention programs with people with disabilities, and were familiar with different data recording methods.

### Technology System: Components

The technology system included a touch screen monitor of 27-48 cm, which was linked to a PC. The computer had internet connection, which allowed for the use of a specific web application. This application controlled the presentation of the leisure options (ie, songs, comic sketches, or cartoons), the cognitive activity (ie, match-to-sample tasks), and the physical exercise (ie, body movements) on the touch screen monitor (see the following section). Moreover, the web application enabled the touch screen monitor to (1) recognize different response configurations (eg, standard input responses, heavy touch, prolonged touch, tap or taps, and scrolling movements) and, thus, (2) react to those responses in a manner consistent with the content of the program [53]. The web application is freely available [54].

### Technology System: Functioning

**Figure 1:**
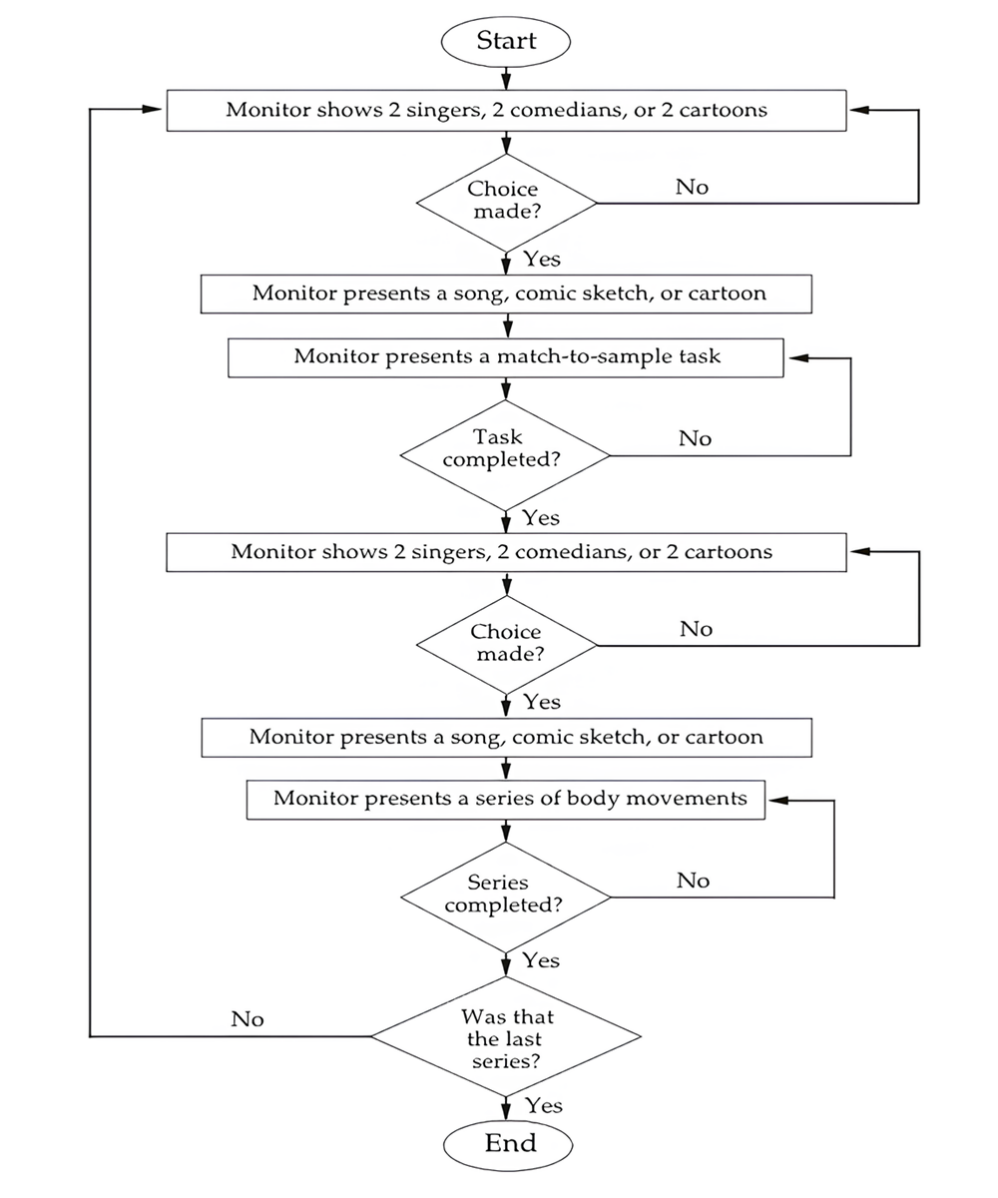
Flowchart summarizing the technology system’s functioning.

At the beginning of a session, the touch screen monitor showed 2 leisure options (ie, the images of 2 singers, 2 comedians, or 2 cartoons depending on the participants’ preferences and hearing condition). The participants could choose 1 of the 2 alternatives by touching it. This caused the touch screen monitor to show a 2-minute video of a song, comic sketch, or cartoon consistent with the choice made by the participants. The only exception to this was Lilah, for whom comic sketches or cartoons lasted 1 minute. Observations indicated that her positive attention to those stimuli tended to decrease after 1 minute. Figure 1 provides a summary of the technology system’s functioning.

**Figure 2:**
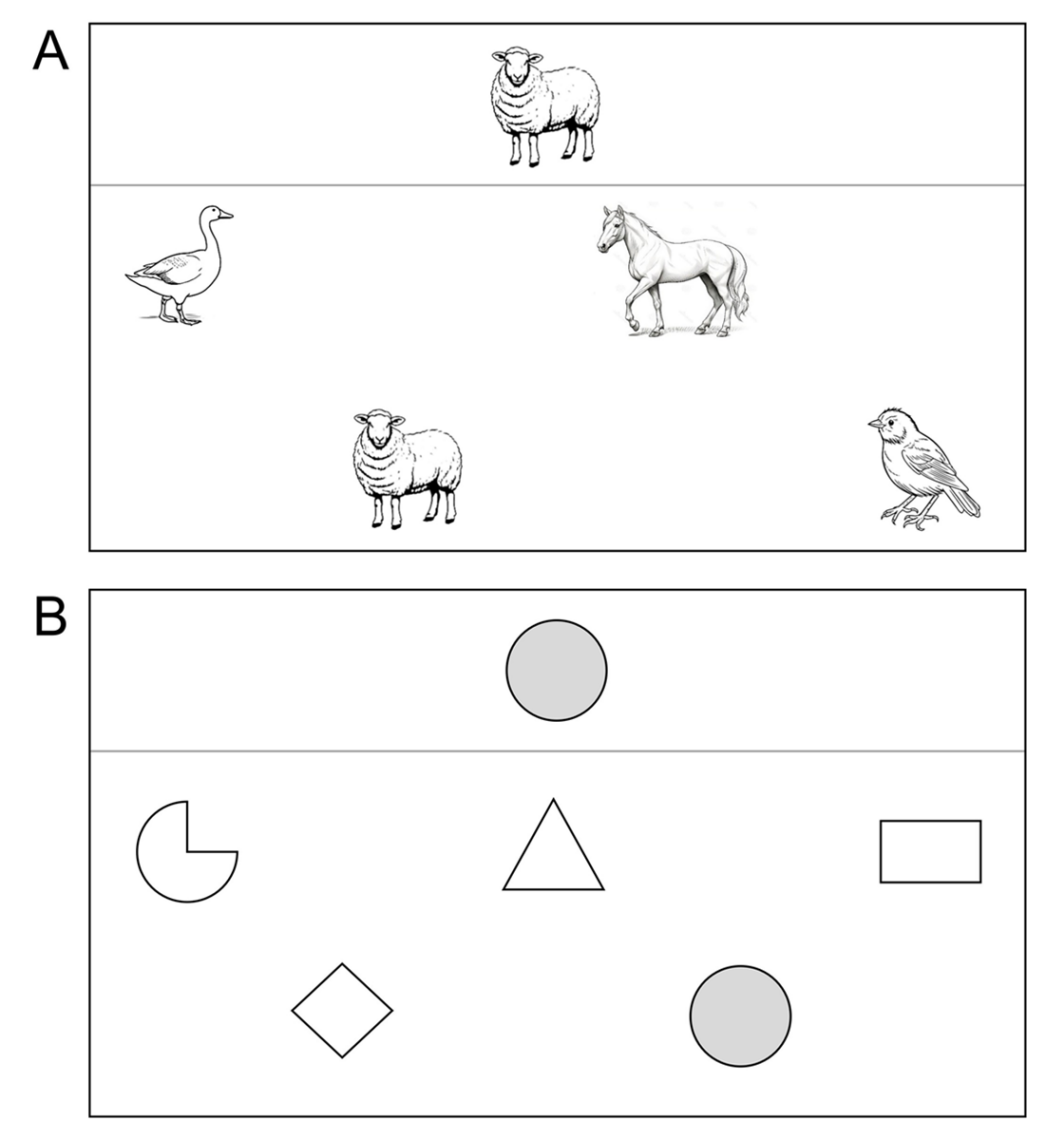
Schematic representation of 2 possible pages (A and B) of a match-to-sample task. Each page shows the sample (top) and the match stimuli available (bottom), which are shown in gray and white in this figure.

At the end of the song, sketch, or cartoon, the touch screen monitor presented a match-to-sample task consisting of 5 or 8 different pages (images), each of which represented a sample stimulus (eg, a colored animal or geometric shape) and 4 other colored stimuli, including the correct match (Figure 2A-B). The pages were presented individually so that the participants could see only 1 page at a time. In each of the pages, the participants were to touch the stimulus that correctly matched the sample. Touching the correct match caused the appearance of a picture showing approval for the response (eg, a woman with thumbs up). This was followed by the appearance of the next page of the task with the next set of stimuli, in which the participants were to identify and touch the correct match. Touching the incorrect match led to the permanence of the same page on the monitor. Progress to the next page occurred only after the participants touched the correct match or a preset interval of 20 to 30 seconds elapsed.

Once the match-to-sample task was completed (ie, with answers provided for each of the 5 or 8 pages), the touch screen monitor showed 2 new leisure options (ie, images of 2 singers, 2 comedians, or 2 cartoons), and all conditions were as those described previously. At the end of the song, sketch, or cartoon, the touch screen monitor presented a video in which the research assistants modeled a series of body movements that the participants were to reproduce. For example, the series could consist of the research assistants standing and sitting 10 or more times or raising their arms and bringing them down to their knees for 10 or more times. The research assistants movement modeling was combined with verbal instructions and encouragements to reproduce those movements. Background color changes (eg, red for arms up and white or green for arms down) could be used for the participants with hearing loss to foster their attention and movement discrimination.

Once the series of body movements had been completed, a new 4-stage sequence (ie, singers, comedians, or cartoons; match-to-sample task; singers, comedians, or cartoons; and body movements) started. The new sequence included different singers, comedians, or cartoons; a different match-to-sample task; and new body movements or new combinations of them. A session included 3 sequences so that the participants had to (1) make 6 choices between singers, comedians, or cartoons (thus accessing 6 songs, 6 comic sketches, or 6 cartoons); (2) complete 3 match-to-sample tasks; and (3) perform 3 series of body movements.

### Data Recording

The measures recorded were (1) the songs, comic sketches, or cartoons accessed correctly and independent of research assistants’ help; (2) the match-to-sample responses carried out correctly (ie, those targeting the correct match in the first attempt) and independent of research assistants’ help; (3) the body movements reproduced correctly and independent of research assistants’ help; (4) the instances of research assistants’ help; and (5) the length of the sessions. The research assistants recorded all 5 measures during the baseline and intervention sessions.

Interrater agreement was checked in 22.4% (15/67; Adriel) to 26.0% (20/77; Jade) of single-participant sessions through the use of a reliability observer. Agreement on songs, comic sketches, or cartoons accessed; correct match-to-sample responses; and instances of research assistants’ help were recorded when the research assistants and the reliability observer had the same scores. Agreement on body movements and session length was recorded when the research assistants and reliability observer had scores that differed by no more than 3 units and 2 minutes, respectively. The percentage of agreement (computed for each participant by dividing the number of sessions in which agreement was reported for all measures by the total number of sessions involving the reliability observer and multiplying this by 100%) was 90% or above for all participants.

### Experimental Conditions and Data Analysis

#### Overview

This study was implemented according to a nonconcurrent multiple baseline design across participants [55,56]. Initially, there was a baseline phase in which the participants were provided with a touch screen monitor that worked in a standard manner. This phase included different numbers of sessions for the participants as required by the design. Two intervention phases were then implemented, during which the participants were provided with the technology system described previously.

To guarantee that the research assistants would implement the sessions accurately (ie, to foster procedural fidelity [57]), familiarization and feedback strategies were used. Familiarization consisted of 2 preliminary meetings in which the research assistants were to practice the baseline and intervention conditions under supervision. Feedback consisted of comments provided to the research assistants after the sessions to inform them as to whether their performance was accurate or needed corrections. Feedback was delivered by a study coordinator who had access to videos of all the sessions.

The baseline and intervention data were reported in graphic format. The percentage of nonoverlapping data (PND), a data analysis method commonly used in single-case experimental research [58], served to determine the impact (effect size) of the intervention on (1) songs, comic sketches, or cartoons accessed; (2) correct match-to-sample responses provided; and (3) body movements reproduced. Specifically, the PND method served to calculate the percentage of intervention sessions with a score higher than the highest baseline score for each measure.

#### Baseline

The baseline phase included 5 to 11 sessions, during which the technology system described previously was not available and the touch screen monitor worked in a standard manner. Initially, the monitor showed the Google icon. Touching (activating) this icon led the monitor to show a web page with 3 separate folders on the favorites bar: one concerning preferred leisure events (ie, songs, comic sketches, or cartoons), a second one concerning match-to-sample tasks, and a third one concerning body movements. Touching (opening) the first folder led to websites containing videos of preferred songs, comic sketches, or cartoons. Touching (opening) the second folder led to websites containing specific match-to-sample tasks. Touching (opening) the third folder led to websites containing videos of different body movements. Initially, the research assistants modeled the response to access a preferred song, comic sketch, or cartoon (depending on the participants), that is, touching the Google icon; touching (opening) the leisure folder; and touching (opening) the website containing a video with a preferred song, comic sketch, or cartoon. The video was stopped shortly thereafter, and the participants were asked to produce the response on their own. If the participants did not manage to do so for 20 to 30 seconds (ie, an interval considered sufficient for them to produce a response), the research assistants repeated the sequence to allow them to access a song, comic sketch, or cartoon for 2 minutes (1 minute in the case of Lilah).

At the end of the song, sketch, or cartoon, the research assistants modeled the response to access a match-to-sample task (ie, cognitive activity). The response consisted of (1) touching the Google icon, which led to the appearance of the leisure, match-to-sample, and body movement folders; (2) touching (opening) the match-to-sample folder, which led to websites containing match-to-sample tasks; and (3) touching (opening) one of these websites showing a specific match-to-sample task. The task remained visible only briefly. The participants were then asked to proceed on their own. If they did not manage to do so for 20 to 30 seconds, the research assistants repeated the sequence to allow them to access a task and helped them (if needed) to provide match-to-sample responses.

After the match-to-sample responses, the research assistants modeled the response for accessing a song, comic sketch, or cartoon. All steps and conditions were identical to those described previously. Once the song, comic sketch, or cartoon had ended, the research assistants modeled the response for accessing the body movement (ie, physical activity) option. The sequence involved (1) touching the Google icon; (2) touching (opening) the body movement folder; and (3) touching (opening) one of the websites, in which a video with 10 repetitions of specific body movements (eg, moving arms up and down) was presented. The video was soon halted, and the participants were asked to proceed on their own. Conditions for research assistants’ help in case of participant failure were as described previously.

The session was interrupted (to reduce frustration) once the participants had received 4 instances of research assistants’ help for opening the websites and accessing songs, comic sketches, or cartoons; match-to-sample tasks; and body movements. The scoring for songs, sketches, or cartoons accessed; match-to-sample responses provided; and body movements performed was negative if the participants required research assistants’ help to open the websites leading to them. Negative scoring was also used in relation to the songs, sketches, or cartoons; match-to-sample responses; and body movements missed due to session interruption.

#### Intervention Phase 1

During the first intervention phase (including 28-40 sessions), the participants were provided with the technology system that worked as described in the Technology System: Functioning section and Figure 1. At the start of a session, the touch screen monitor showed the pictures of 2 leisure stimuli, and the participants were to touch one of them to start a song, comic sketch, or cartoon depending on the pictures displayed. A song, comic sketch, or cartoon lasted 2 minutes (1 minute in the case of Lilah). Thereafter, the touch screen monitor showed (one at a time) the 5 pages of the first match-to-sample task available. For each page, the participants were to touch the stimulus that matched the sample. Once the participants had completed the match-to-sample task, the touch screen monitor presented 2 new leisure stimuli. The participants were to choose one of them as mentioned previously. At the end of the leisure event (ie, song, comic sketch, or cartoon), the touch screen monitor presented a video with the research assistants showing a series of 10 to 18 body movements. The participants were to imitate (reproduce) those movements. Completion of the body movement series led to the beginning of a new 4-stage sequence (ie, leisure stimuli, match-to-sample task, leisure stimuli, and body movements). A session included 3 of those sequences, which differed in terms of the leisure events, match-to-sample tasks, and body movements presented.

The phase was introduced by 4 to 6 practice sessions in which the participants were provided with research assistants’ help to manage their access to leisure events, responses to the match-to-sample tasks, and imitation (reproduction) of body movements. During the regular sessions that followed, research assistants’ help would only be available if the participants failed to access a leisure event for 20 to 30 seconds.

#### Intervention Phase 2

During the second intervention phase (including 33-47 sessions), all conditions were as in intervention phase 1 except that (1) the match-to-sample tasks included 8 (rather than 5) pages and (2) the series of body movements included 14 to 30 (rather than 10-18) movements.

## Results

**Figure 3:**
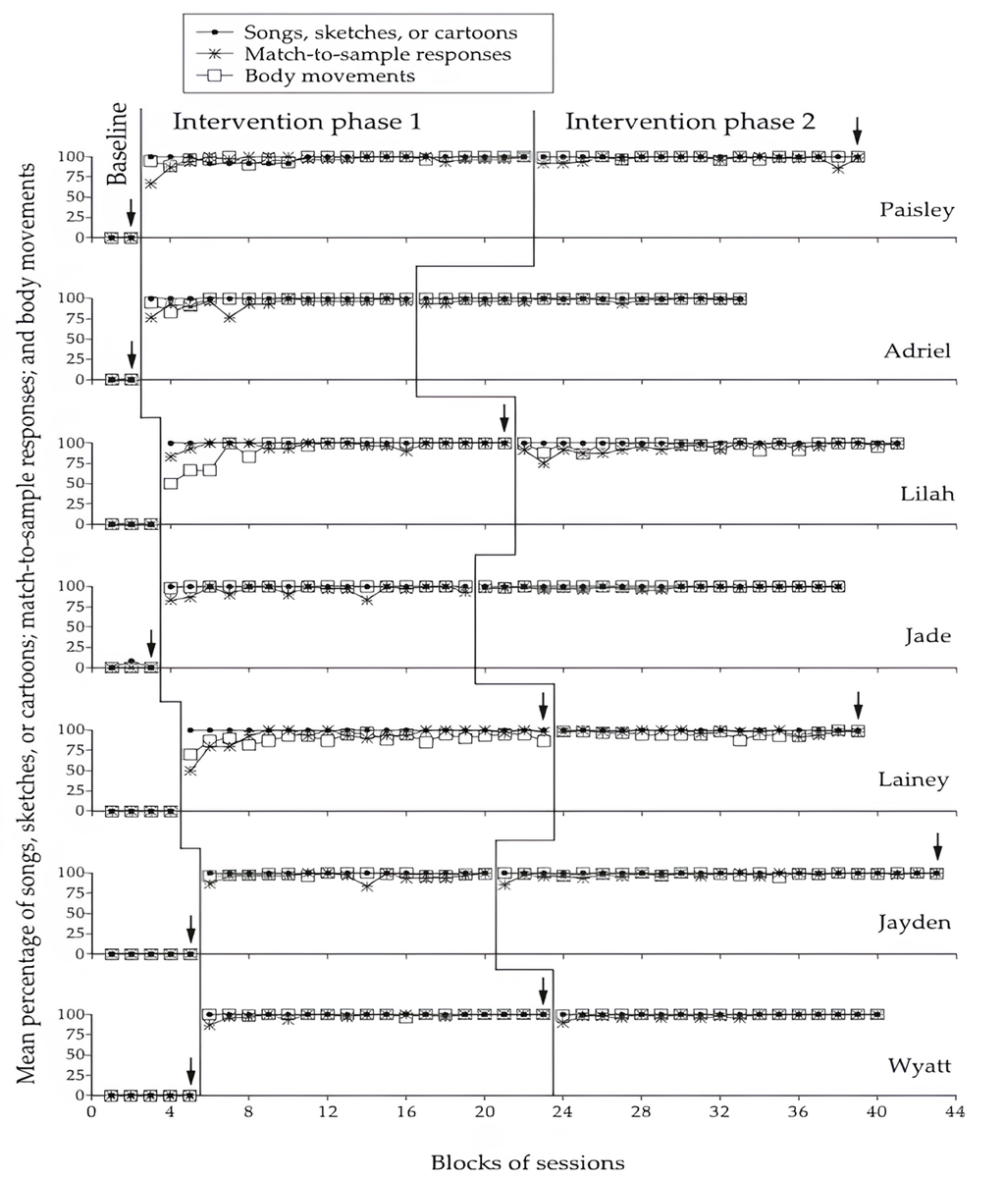
Dots, asterisks, and empty squares represent the mean percentage of songs, comic sketches, or cartoons accessed; match-to-sample responses provided; and body movements performed over blocks of 2 sessions, respectively. Blocks of 3 sessions are marked with an arrow.

Figure 3 summarizes the baseline and intervention data for the 7 participants. The dots represent the mean percentage of songs, comic sketches, or cartoons accessed independent of research assistants’ help per session over blocks of 2 sessions. The asterisks and the empty squares represent the mean percentage of correct match-to-sample responses provided and correct body movements performed per session over the same blocks of sessions, respectively. The arrows mark blocks of 3 sessions that took place at the end of the baseline and intervention phases. The practice (familiarization) sessions preceding the start of intervention phase 1 do not appear in the figure.

During the baseline phase, the mean percentage of songs, comic sketches, or cartoons accessed; match-to-sample responses provided; and body movements performed correctly and independent of research assistants’ help was 0% for all participants except Jade. She managed to access 1 song throughout the 7 baseline sessions available to her. Participants were largely characterized by inactivity and failed to produce effective touch screen responses on those occasions in which they took the initiative to follow the research assistants’ modeling on how to activate the Google icon and open the 3 basic folders and the related websites. The aforementioned inactivity and difficulties (causing dependence on research assistants’ help) led to the interruption of all baseline sessions. The length of those sessions was always below 15 minutes.

During intervention phase 1, the mean percentage for songs, comic sketches, or cartoons accessed correctly and independent of research assistants’ help per session was 100% for all participants except Paisley, whose mean percentage was 97.9% (SD 3.7%). The mean percentage of correct match-to-sample responses provided and correct body movements performed independent of the research assistants’ help ranged between 92.7% (SD 12.1%; Lainey) and 98.2% (SD 3.5%; Wyatt) and between 90.1% (SD 6.5%; Lainey) and 99.9% (SD 0.4%; Jade), respectively. The mean session length varied between slightly over 15 minutes (Lilah) and 23 minutes (Lainey).

During intervention phase 2, the mean percentage for songs, comic sketches, or cartoons accessed correctly and independent of the research assistants’ help per session was 100% for all participants. The mean percentage for correct match-to-sample responses provided and correct body movements performed independent of the research assistants’ help ranged between 94.2% (SD 6.0%; Lilah) and 98.6% (SD 1.7%; Jade) and between 95.6% (SD 3.1%; Lainey) and 100% (SD 0%; Adriel), respectively. The mean session length varied between approximately 16.5 minutes (Lilah) and 27.5 minutes (Lainey).

The PND method showed an index of 1 on each of the aforementioned 3 measures for all participants. Indeed, the data values of the intervention sessions were always higher than the baseline data values on each of the measures. This evidence confirms the strong impact of the intervention with the technology system across participants and measures.

## Discussion

### Principal Findings

The results suggest that participants with intellectual or intellectual and sensory-motor disabilities could use the technology system introduced in this study to access songs, comic sketches, or cartoons; provide correct match-to-sample responses; and perform correct body movements independent of research assistants’ help. These results confirm previous findings showing that technology-aided programs can be set up to help people with intellectual and multiple disabilities engage in functional activities independent of staff [3,7,11,41]. These results also extend previous evidence. In fact, this study (1) included a form of cognitive (match-to-sample) activity and basic physical exercise adjustable to people with intellectual and multiple disabilities and (2) introduced a new, modified touch screen system. In light of the aforementioned results, several considerations may be in order.

First, enabling people with intellectual and multiple disabilities to remain constructively busy for practically relevant time periods independent of staff supervision may be considered a meaningful achievement for the participants and their rehabilitation context. Indeed, participants can develop and strengthen self-determination and initiative, reach a higher (more satisfactory) level of functioning, and experience a better quality of life in line with their rights [59-64]. The rehabilitation context may be able to include extra opportunities for constructive engagement in the participants’ daily schedules without significant investment of staff time [7,65,66].

Second, access to preferred leisure stimuli may be considered important for the participants’ enjoyment and possibly their level of satisfaction with the sessions [50,67]. Engaging in cognitive activity and physical exercise may be important for the participants’ intellectual and physical stimulation and contribute to their rehabilitation progress and well-being [11,14,68,69]. Combining (interspersing) access to preferred leisure stimuli with cognitive and physical activities, as was done in this study, might help (motivate) the participants to constructively engage in the latter types of activities as well irrespective of the fact that these activities may not necessarily be preferred by them [49,50]. While no formal data are available to confirm the accuracy of the aforementioned statement, the participants’ positive and stable performance throughout the intervention sessions may be taken to suggest that they remained motivated to engage in each of the activities available, thus making their involvement in the study constructive and profitable.

Third, the participants’ motivation and positive performance during the intervention phase may be ascribed not only to the presence of preferred leisure events among the options available but also to other factors. For example, the cognitive (match-to-sample) tasks and physical exercises (body movements) were tailored (adjusted) to their skill level in terms of the stimuli and movements included, thus minimizing difficulties, failures, and frustration. The participants received positive feedback for the cognitive (match-to-sample) responses completed, and it is possible that such feedback represented a mild form of reinforcement [50]. The body movements were supported by instructions and encouragements for the participants with typical hearing and were cued (emphasized) through background color changes for the participants with hearing loss. The touch screen monitor reacted promptly and consistently to the participants’ touch responses, minimizing any risk of failures.

Fourth, the web application included in the technology system ensured that the participants could make their touch responses successful (ie, choosing and activating a preferred leisure stimulus and selecting a matching stimulus) regardless of the accuracy (quality) of their contact with the touch screen monitor. Specifically, they could be successful irrespective of whether their contact consisted of a standard input touch, a heavy touch, a prolonged touch, taps, or scrolling movements. The use of such an application was considered critically important (necessary) based on the preliminary assessment of the participants’ response characteristics and their baseline performance. Whether the application was still strictly necessary for all participants by the end of the study is not clear as no data were collected on that. Informal observations seemed to suggest that the participants’ response schemes did not show particular changes over time.

### Limitations and Future Research

The 3 main limitations of this study involve the lack of assessment of participants’ satisfaction with the touch screen technology system and the intervention sessions, the lack of social validation of the program and the related technology, and the absence of maintenance and generalization data on the effects of the program. Regarding the participants’ satisfaction, 2 basic points can be made. First, the fact that the participants remained constructively engaged throughout the intervention sessions seems to suggest that they were comfortable (and possibly satisfied) with the sessions and their content [49,50,67]. Moreover, anecdotal reports provided additional support for this view. Second, future studies are expected to include a formal assessment of participant satisfaction. The assessment could involve two approaches: (1) measuring the participants’ indices of happiness during and outside of the sessions and (2) having the participants choose between the sessions and other types of daily occupation [70,71].

The lack of social validation of the program and the technology used can be amended by future studies through surveys of staff and caregivers on these points. In practice, staff and caregivers could be (1) shown videos of the program (intervention) sessions with the participants using the technology and (2) asked to rate the program and technology in terms of impact, user-friendliness, and usability [72-74]. The lack of maintenance and generalization data can be amended by (1) extending the data collection period to verify whether the participants’ performance remains stable over time and (2) implementing the program across different settings to ascertain whether the participants continue to be successful irrespective of the context [50,75].

The lack of assessment of whether the cognitive activity and physical exercise had an impact on the participants’ functioning in those areas may be considered a fourth limitation of this study. Future research may need to address this limitation and find ways (ie, identify appropriate measures) to carry out such an assessment. The relatively small number of participants might not be considered an additional limitation as far as the internal validity of the data reported is concerned. In fact, the single-case research design used in this study is admittedly adequate to ensure such validity [55,56,76]. To test the external validity of those data, one would need to implement single-case replication studies and possibly group studies including experimental and control conditions [77-79].

### Conclusions

The results suggest that the intervention program with the support of the technology system was effective in enabling participants with intellectual and multiple disabilities to independently access leisure events, manage match-to-sample tasks, and perform series of body movements. These findings can be considered encouraging as to the possibility of helping people with serious disabilities reach relevant levels of independent occupation through technology-aided programs. However, no conclusions about their implications can be drawn given the limitations of this study discussed previously. New research will need to counter those limitations and assess the generality and robustness of these data. New research might also seek to upgrade the technology system to make it simpler and more easily available.

## Abbreviations

PND: percentage of nonoverlapping data

## Data Availability

The datasets generated during this study are available on OneDrive repository [80]
